# Vero CCL-81 and Calu-3 Cell Lines as Alternative Hosts for Isolation and Propagation of SARS-CoV-2 Isolated in Malaysia

**DOI:** 10.3390/biomedicines11061658

**Published:** 2023-06-07

**Authors:** Siti Nur Zawani Rosli, Sitti Rahmawati Dimeng, Farah Shamsuddin, Mohammad Ridhuan Mohd Ali, Nur Afrina Muhamad Hendri, Jeyanthi Suppiah, Rozainanee Mohd Zain, Ravindran Thayan, Norazah Ahmad

**Affiliations:** 1 Bacteriology Unit, Infectious Disease Research Center, Institute for Medical Research, National Institutes of Health, Ministry of Health Malaysia, 40170 Setia Alam, Malaysia; 2 Electron Microscopy Unit, Special Resource Center, Institute for Medical Research, National Institutes of Health, Ministry of Health Malaysia, 40170 Setia Alam, Malaysia; 3 Virology Unit, Infectious Disease Research Center, Institute for Medical Research, National Institutes of Health, Ministry of Health Malaysia, 40170 Setia Alam, Malaysia; 4 Infectious Disease Research Center, Institute for Medical Research, National Institutes of Health, Ministry of Health Malaysia, 40170 Setia Alam, Malaysia

**Keywords:** SARS-CoV-2 isolation, COVID-19, coronavirus

## Abstract

Severe acute respiratory syndrome coronavirus 2 (SARS-CoV-2) has been identified as the etiologic agent for the pneumonia outbreak that started in early December 2019 in Wuhan City, Hubei Province, China. To date, coronavirus disease (COVID-19) has caused almost 6 million deaths worldwide. The ability to propagate the virus into a customizable volume will enable better research on COVID-19 therapy, vaccine development, and many others. In the search for the most efficient replication host, we inoculated three (3) local SARS-CoV-2 isolates of different lineages (Clade L/Lineage B Wuhan, Clade GR/Lineage B.1.1.354, and Clade O/Lineage B.6.2) into various clinically important mammalian cell lines. The replication profile of these isolates was evaluated based on the formation of cytopathic effects (CPE), viral load (Ct value and plaque-forming unit (pfu)), as well as observation by electron microscopy (EM). Next-generation sequencing (NGS) was performed to examine the genomic stability of the propagated SARS-CoV-2 in these cell lines. Our study found that Vero E6 and Vero CCL-81 cell lines posed similar capacities in propagating the local isolates, with Vero CCL-81 demonstrating exceptional potency in conserving the genomic stability of the Lineage B Wuhan isolate. In addition, our study demonstrated the utility of Calu-3 cells as a replication host for SARS-CoV-2 without causing substantial cellular senescence. In conclusion, this study provides crucial information on the growth profile of Malaysian SARS-CoV-2 in various mammalian cell lines and thus will be a great source of reference for better isolation and propagation of the SARS-CoV-2 virus isolated in Malaysia.

## 1. Introduction

A novel coronavirus (nCoV) has been responsible for the recent pneumonia outbreak that started in early December 2019 in Wuhan City, Hubei Province, China [1]. The disease spread rapidly around the world, affecting all continents. As a result, the WHO declared the outbreak a global pandemic on 11 March 2020 [2,3]. After 3 years, more than 600 million confirmed coronavirus disease 2019 (COVID-19) cases worldwide have been recorded and accounted for more than 6 million deaths. In Malaysia, the first case of COVID-19 was reported in January 2020 among visitors from China who entered Malaysia via Singapore. Since then, Malaysia has dealt with three major waves of the COVID-19 outbreak, with more than 36,000 fatalities [4,5].

During the initial pandemic, the *Coronavidae* Study Group (CSG) of the International Committee on Taxonomy of Viruses recognized the novel coronavirus as the forming sister clade to the prototype human and bat severe acute respiratory syndrome (SARS) coronavirus and designated it as Severe Acute Respiratory Syndrome Coronavirus 2 (SARS-CoV-2) [6]. This virus is reported to contain 380 amino acid substitutions in comparison to SARS or SARS-like coronaviruses, which has caused increased functional and pathogenic divergence of this virus [6]. Cellular entry of coronavirus into host cells is highly dependent on the binding of spike protein (S) to a specific cellular receptor and subsequent priming by cellular protease. For SARS-CoV-2, it employs angiotensin converting enzyme-2 (ACE2) as the main receptor to facilitate their entry into cells, with priming by transmembrane serine protease 2 (TMPRSS2) [7,8,9]. ACE2 is abundantly expressed in the heart, kidneys, testes, and lungs [10]. Therefore, these tissues could easily be the target for the SARS-CoV-2 infection.

To date, many studies carried out on COVID-19 that focused on detection and therapies have been hampered by the limited capacity of the virus to propagate, albeit this step is the key to understanding virus pathogenicity. Therefore, this study aims to characterize the replication profile and cell culture tropism of this virus in different mammalian cell lines, which eventually will enable researchers to propagate this virus in large quantities with minimal viral genetic mutations.

## 2. Materials and Methods

### 2.1. Virus

Three virus isolates (WC1114/20, WI119/20, and WC81849/20) were obtained from the archive of the Virology Unit, Institute for Medical Research (IMR). The identity of isolates was previously confirmed by real-time reverse transcription (RT-PCR) targeting the spike (S), envelope (E), and nucleocapsid (N) genes of SARS-CoV-2 and whole genome sequencing. The samples were identified as clades L/Lineage B Wuhan (WC1114/20), GR/Lineage B.1.1.354 (WI119/20), and O/Lineage B.6.2 (WC81849). The initial culture for each virus stock of SARS-CoV-2 was performed in African green monkey cells (Vero E6; ATCC^®^ CRL-1586™) until passage 4. Virus quantification was performed using plaque assays to determine the multiplicity of infection (MOI) for the study, and a MOI of 1 was chosen for all the assays. To achieve MOI = 1, 1 × 10^6^ pfu/mL of virus were inoculated into 1 × 10^6^ cells (70% confluency). 

All inoculations involving SARS-CoV-2 were carried out under biosafety level 3 (BSL-3) containment conditions. 

### 2.2. Cell Lines and Cell Cultures

Vero E6 cells, Vero CCL-81 cells (ATCC^®^ CCL-81™), and human lung carcinoma cells (A549; ATCC^®^ CRL-185™) were cultured in Dulbecco’s minimal essential medium (DMEM; Gibco, Grand Island, NE, USA) supplemented with HEPES (Sigma Aldrich, St. Louis, MO, USA), 10% heat-inactivated fetal bovine serum (FBS; Gibco, Paisley, UK), and penicillin-streptomycin (10,000 U/mL) (Gibco, Grand Island, NE, USA). Human fibroblast cells (MRC-5; ATCC^®^ CRL-171™) and human airway epithelial cells (Calu-3; ATCC^®^ HTB-55™) were maintained in Eagle’s minimum essential medium (EMEM; ATCC, Manassas, VA, USA) supplemented with 10% heat-inactivated FBS and penicillin-streptomycin. The cells were passaged every 2 to 3 days by dissociating them with TrypLE™ Express Enzyme (Gibco, Grand Island, NE, USA). All cells were grown in a humidified 37 °C incubator with 5% CO_2_.

### 2.3. Multiple Mammalian Cell Line Assay

Cell lines were seeded into 6-well plates at 5.0 × 10^5^ cells/mL in their respective growth mediums, 24 h before infection. The cells were inoculated with SARS-CoV-2 at a multiplicity of infection (MOI) of 1 for 30 min, with occasional shaking every 5 to 10 min. Non-attached virus was removed by washing the cells with phosphate-buffered saline (PBS; Gibco, Paisley, UK). Then, the respective medium supplemented with 2% FBS and penicillin-streptomycin was added to the total of 2 mL/well. The presence of SARS-CoV-2-specific cytopathic effects (CPE) was observed daily for 7 days. The supernatant and cell lysate were collected daily by scraping, centrifuged at 200× *g* for 5 min, and stored at −80 °C. 

### 2.4. RNA Extraction and Quantitative Reverse Transcription–Polymerase Chain Reaction (rt-qPCR)

The cultures were collected daily from day 0 until day 7 to examine the presence of the virus genome in the culture supernatant. The presence of SARS-CoV-2 in the cell lines was examined by RT-qPCR using the BGI’s real-time fluorescent RT-PCR kit, according to the manufacturer’s protocol. This kit evaluated the presence of the virus genome based on the nucleic acid amplification of ORF1ab and the N gene of SARS-CoV-2. 

Total RNA from the supernatant was extracted using the QIAamp viral RNA Mini Kit (Qiagen, Hilden, Germany) according to the manufacturer’s instructions.

### 2.5. Plaque Assay

Supernatants collected from infected cell lines were used for plaque assays on Vero E6 cells. The cells were seeded into 6-well plates at 1 × 10^6^ cells/mL 24 h before infection. Supernatants were diluted in 10-fold serial dilutions in Hanks’ Balanced Salt Solution (HBSS; Gibco, Bleiswijk, The Netherlands) supplemented with 0.04% bovine serum albumin (BSA; Sigma Aldrich, St. Louis, MO, USA). Then, 300 μL of each dilution was added to the cell monolayer and left for adsorption for 30 min with shaking at 5 to 10 min intervals. The inoculum was aspirated, and the monolayer was then overlaid with 3 mL of 1.2% agarose (Invitrogen, Carlsbad, CA, USA) in 2x DMEM supplemented with 20% FBS. Plates were incubated for 3 days before fixation using 50% trichloroacetic acid (TCA; Sigma Aldrich, Steinheim, Germany). The agarose plug was then removed after 2 h of fixation, washed with water, and stained with 1% crystal violet (Sigma Aldrich, St. Louis, MO, USA) in 20% ethanol. Plaques were quantified and recorded as plaque-forming units per unit volume (PFU/mL).

### 2.6. Transmission Electron Microscopy

For electron microscopy, the cell lysate was inactivated using 2.5% glutaraldehyde in 0.1 N PBS for 1 h at room temperature, followed by centrifugation at 1000× *g* for 3 min. The cell pellet was rinsed with Milli-Q water. After low-speed centrifugation, the pellet was post-fixed in 1% osmium tetroxide for 1 h at room temperature, followed by en bloc staining using EM Zero stain for 5 min. Then, the pellet was subjected to a dehydration process with a graded series of acetone dilutions. Next, the pellet was transitioned into infiltration with epoxy resin/100% acetone in a 1:1 mixture before embedding in epoxy resin. Embedded samples were polymerized at 60 °C overnight before sectioning by the EM UC7 ultramicrotome (Leica, Wetzlar, Germany) model. Subsequently, sections of 70 nm were transferred to copper 200-mesh electron microscopy grids. The ultrathin section grid was stained with Reynold’s stain and examined using an Tecnai™ G2 Spirit Twin (Thermofisher Scientific, Waltham, MA, USA) operating at an acceleration voltage of 120 kV. Electron micrographs were collected using a Gatan Orius SC1000B 200Kv CCD (Gatan, CA, USA) camera. Virion diameter and glycoprotein features were measured and photographed.

### 2.7. Whole Genome Sequencing

The most suitable cell lines to support multiplication of the SARS-CoV-2 isolate were determined by the genomic stability of the isolate in different cell lines. For sample preparation and sequencing of the SARS-CoV-2 genome, viral RNA was isolated from infected culture supernatant using the QIAamp Viral RNA Mini Kit (Qiagen, Hilden, Germany). The detection of viral RNA was carried out by RT-qPCR using BGI’s Real-Time Fluorescence RT-PCR Kit (BGI, Wuhan, China), following the manufacturer’s instructions to detect the presence of ORF1ab and the N gene of SARS-CoV-2. Total RNA concentration was quantified using the Thermo Scientific™ Multiskan Sky Microplate Spectrophotometer (Thermo Fisher Scientific, Waltham, MA, USA). Samples that showed the lowest cycle threshold (Ct) value with a total RNA concentration of more than 20 ng/μL without the RNA carrier from each cell line were selected for whole genome sequencing. 

The RNA was amplified with primers for full-length analysis. Next-generation sequencing (NGS) analysis was performed by the Illumina Miseq (Illumina, San Diego, CA, USA). The sequencing was performed by Apical Scientific, Seri Kembangan, Malaysia.

For QC, Illumina raw reads were first removed from the ARTIC V4 primer sequences using bbduk of the BBTools Packages (https://jgi.doe.gov/data-and-tools/bbtools/ (accessed on 1 January 2022)). QC-ed reads were mapped to the reference genome (Severe Acute Respiratory Syndrome Coronavirus 2 isolate Wuhan-Hu-1; MN908947.3) using Bowtie2 (2) with end-to-end alignment type and medium/fast sensitivity. Variant calling was performed using Geneious Prime (3) with a minimum coverage of 100 and standard translation code (Table 1) to infer amino acid change and protein effect. 

Illumina reads were assembled de novo using Spades v3.13 (4) and polished using Pilon v1.23 (5), implemented in Unicycler 0.4.8 (6). Four data sets (WC1114/20 in A549, WC1114/20 in MRC5, WC1114/20 in Vero E6, and WI119/20 in Vero E6) were assembled separately into a single contig. All other data sets yielded 2–3 contigs.

### 2.8. Statistical Methods

All the results were plotted as the mean ± standard error of the mean (SEM). A student t-test and one-way ANOVA test were performed to compare the differences between groups.

## 3. Results

### 3.1. Morphology of Various Mammalian Cell Lines Infected with Malaysian SARS-CoV-2 Isolates

**Figure 1:**
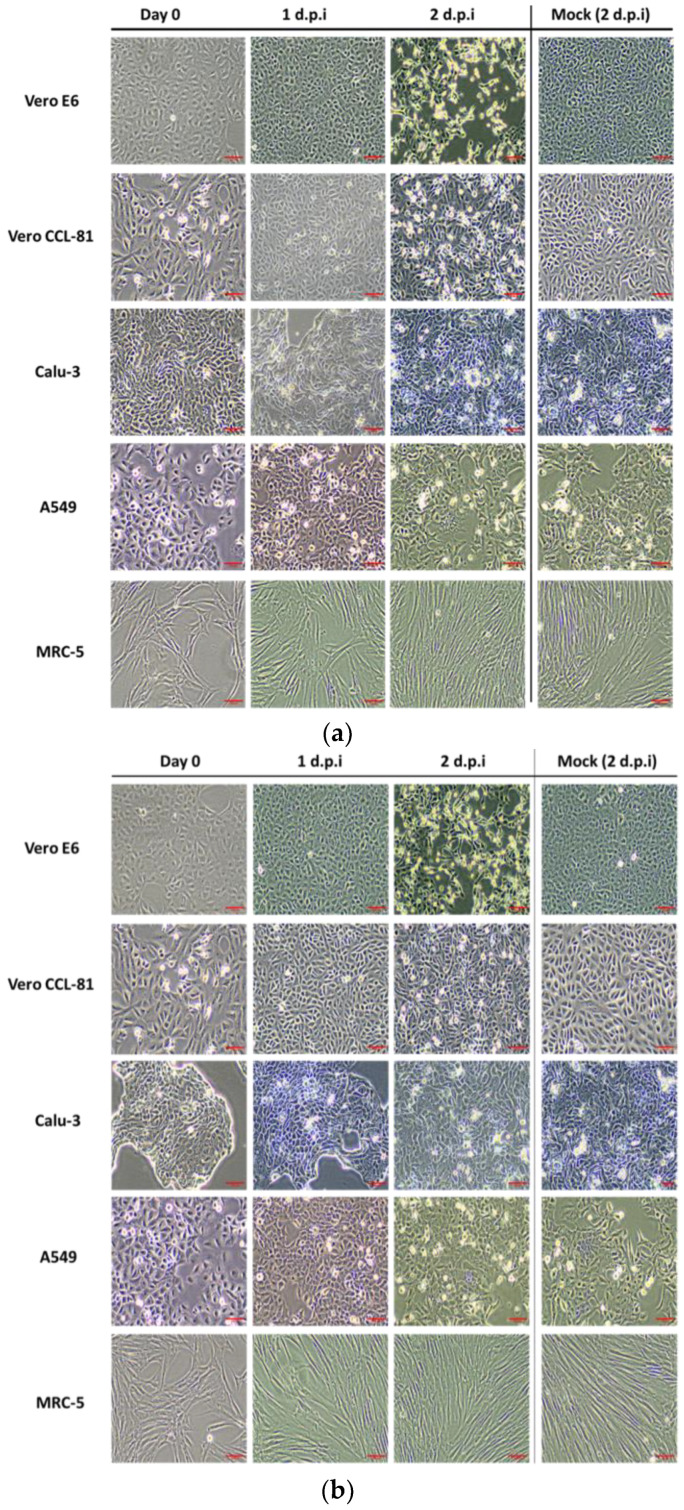

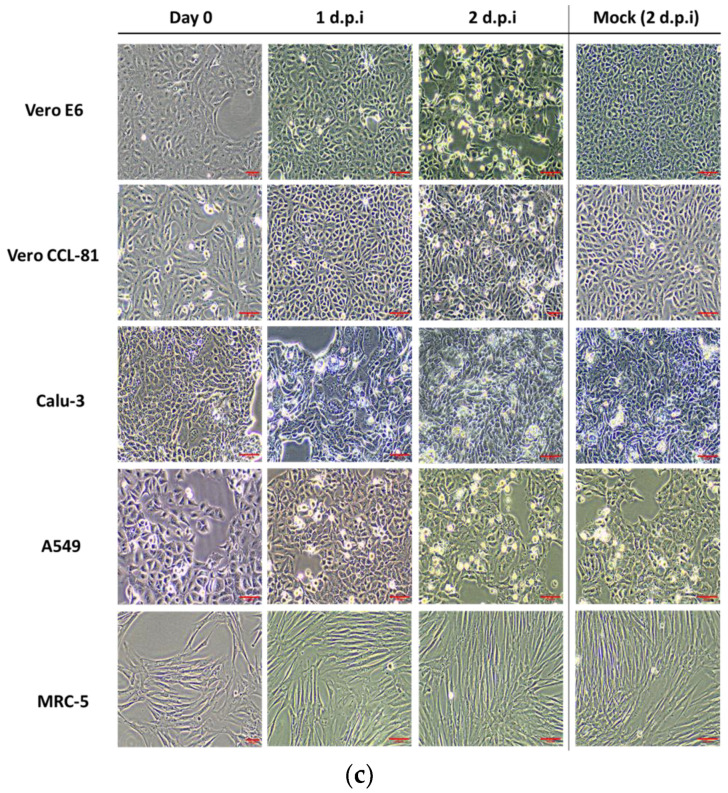
Vero E6, Vero CCL-81, Calu-3, A549, and MRC-5 monolayers at day 0 until day 2 post-infection. Cytopathic effect (CPE) images of these cells inoculated with (**a**) WC1114/20; (**b**) WI119/20; and (**c**) WC81849/20. All cells were inoculated with different SARS-CoV-2 isolates at a MOI of 1. CPE was observed and recorded daily until 7 days post-inoculation (d.p.i) or until cells reached 80% CPE. The images shown are at 10× magnification. The scale bar indicates 500 µm. These experiments were repeated independently three times, with triplicates for each condition.

Of the five tested cell lines, Vero E6 and Vero CCL-81 exhibited CPE in the form of cell lysis against all three virus isolates. The formation of CPE was apparent on day 2 post-inoculation (Figure 1a–c). The other cell lines (A549, MRC, and Calu3) did not exhibit any form of CPE until day 7 of inoculation.

### 3.2. Growth Profile of Malaysian SARS-CoV-2 Propagated in Various Mammalian Cell Lines

**Figure 2:**
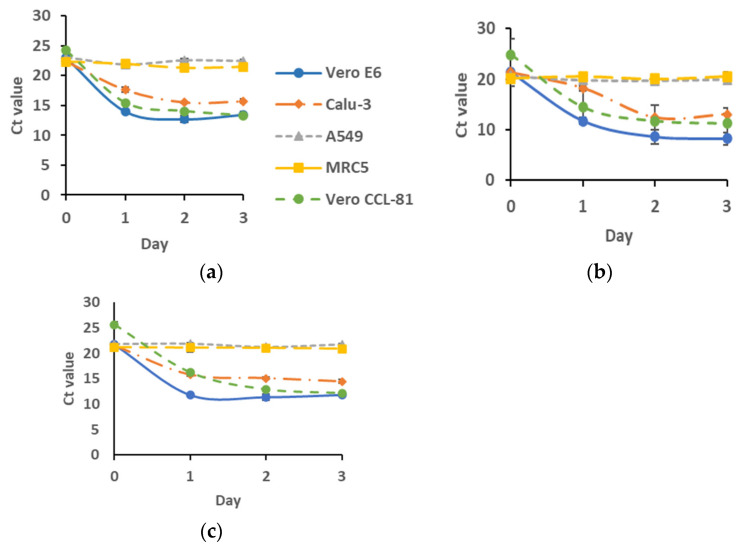
Viral RNA multiplication of (**a**) WC1114/20; (**b**) WI119/20; and (**c**) WC81849/20 in various cell lines at 0-, 1-, 2-, and 3-days post-inoculation. Cells were infected with the virus at a MOI of 1. The viral multiplication of these cell lines was then recorded as the Ct value of ORF1ab. *n* = 3.

Figure 2 shows the Ct values of all the viruses grown in different cell lines. Among the five cell lines studied, viruses grown in Vero CCL-81, Vero E6, and Calu-3 recorded Ct values that were considerably low, which suggested the propagation of the virus in these cell lines. Similar observations were seen in all three isolates used in the study. Meanwhile, cultures that were grown in MRC5 and A549 did not show any progress from day 0 of incubation (Figure 2). Thus, this observation suggests that A549 and MRC5 did not support the growth of these isolates. 

### 3.3. Plaque Formation by Malaysian SARS-CoV-2 Propagated in Various Mammalian Cell Lines

**Figure 3:**
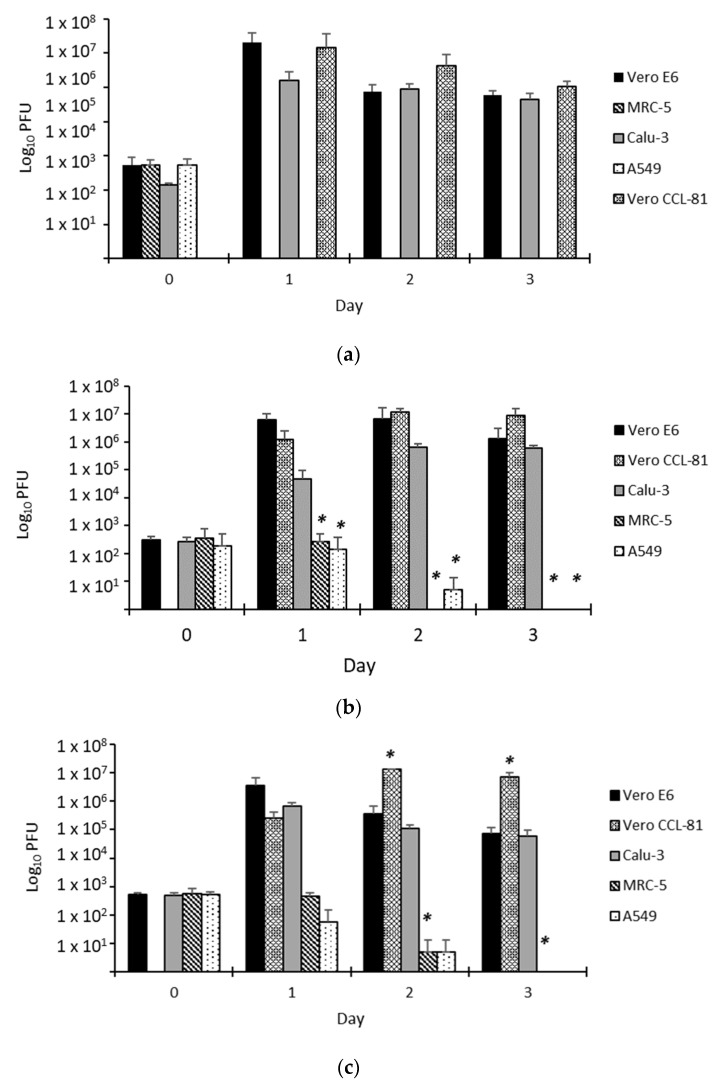

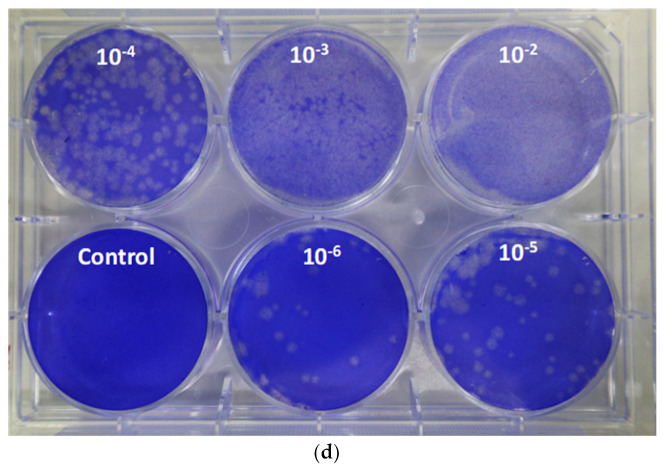
Viral quantitation for (**a**) WC1114/20; (**b**) WI119/20; and (**c**) WC81849/20 in various cell lines. The viral titer is quantitated by plaque assay on Vero E6 cells from day 0 to day 3 post-inoculation. (**d**) Plaque morphology for the virus on Vero E6 at various dilutions. *n* = 3, * *p* < 0.05. A growth comparison was made against the growth in Vero E6. The supernatants used for this assay were also used in the qPCR assay. Some samples did not form detectable plaques at certain time points, i.e., (i) Vero CCL-81 on day 0 (all isolates), (ii) WC1114/20 inoculated on MRC-5 and A549 from day 1 to 3, (iii) WI119/20 and WC81849/20 inoculated in MRC-5 and A549 (day 3), (iv) WI119/20 inoculated on MRC-5 (day 2).

Vero E6 and Vero CCL-81 recorded the highest virus titers. Multiplication of our isolates was encouraging in these cell lines, whereby the virus titer increased rapidly 24 h post inoculation to reach a titer of >10^6^ pfu/mL (Figure 3a–c). In addition to Vero cell lines, our study suggested the capacity of Calu-3 to support the growth of SARS-CoV-2. From the data, our isolates that were grown in Calu-3 did multiply, albeit modestly, to reach a titer of around 10^5^ pfu/mL 48 h post-inoculation. Although we did not see CPE formation on Calu-3, our analysis suggested there was no significant difference between these three cell lines in supporting the multiplication of Malaysian SARS-CoV-2. Conversely, this assay further concluded that MRC-5 and A549 could not support the propagation of Malaysian SARS-CoV-2 (Figure 3a–c).

In addition, our analysis revealed that isolates WI119/20 (Lineage B Wuhan) and WC81849/20 (Lineage B.6.2) demonstrated the highest propagation rate among other isolates under study (Figure 3d).

### 3.4. Analysis of the Structure of the Virus by Electron Micrographs

**Figure 4:**
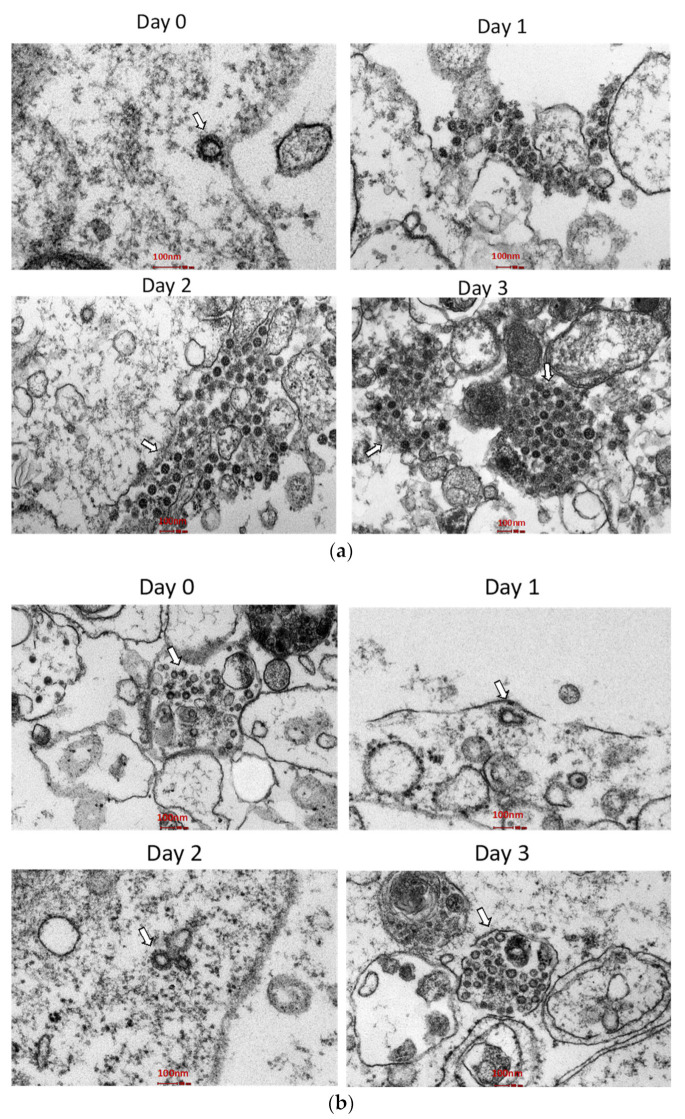
Thin section electron micrographs of (**a**) Vero E6 cells and (**b**) Calu-3 cells, infected with the WI119/20 isolate. Cells were collected soon after virus inoculation (day 0), as well as 24 h (day 1), 48 h (day 2), and 72 h (day 3) after infection for examination by electron microscopy. Arrows point to aggregates of intracellular virions.

Virus particles were visualized in the cell lysates. Images obtained from an electron microscope revealed the coronavirus-specific morphology of distinctive spikes with sizes ranging from 70 to 100 nm. Apart from being highly enfolded in vesicles, virus particles were also observed in a wide range of intracellular organelles (Figure 4a,b).

### 3.5. Full-Length Genome Analysis for the Single Nucleotide Polymorphism (SNP) Analysis

Table 1 shows the mapping statistics. From the analysis, SNPs were detected in all samples except for isolate WC1114/20 inoculated in Vero CCL-81. The sequence data are not shared online.

## 4. Discussion

The selection of cell lines in this study was based on their clinical relevance in the diagnosis and isolation of human viruses, respiratory viruses. Previously, these cells have been instrumental in the isolation of many other respiratory viruses [11,12,13]. The inability to synthesize interferon and abundance of ACE2 expression [13] have provided Vero cells with a convenient avenue and substrate for the propagation of many viruses [14,15]. As reported elsewhere [16,17], our SARS-CoV-2 isolates showed rapid propagation in both lineages of Vero cells, i.e., Vero E6 and Vero CCL-81 (Figure 1). Inoculation of Malaysian SARS-CoV-2 in these cells resulted in the formation of a cytopathic effect (CPE) as soon as 24 h post-inoculation and reached around 70–80% CPE at 48 h post-inoculation, with highly distinctive cell lysis morphology in Vero E6 cells. This observation was supported by data from real-time RT-PCR and plaque assays (Figure 2 and Figure 3). Our analysis concluded that there was no significant difference between Vero E6 and Vero CCL-81 in their susceptibility to infection by the SARS-CoV-2 isolates used in the study. 

Although Vero cells are proven to be useful to propagate the virus, the cells are not suitable for investigation of the pathological mechanisms of host cell response to virus infection. Factors such as the lack of the Type I Interferon genes make the cells highly permissible to virus infection [18]. Consequently, the formation of CPE will be extremely rapid, making it difficult to use these cells in such a study. In addition, Vero cells are derived from monkeys, therefore making them less favorable to be used in the study of human pathophysiology. Considering all these factors, we included three human-derived cell lines in our investigation. Our investigation concluded that Malaysian SARS-CoV-2 isolates did not propagate well in human-derived lung cell lines except for Calu-3 (Figure 2 and Figure 3). Furthermore, we found that Calu-3 has a comparable capacity to Vero cell lines in propagating the virus without causing cell senescence (Figure 1), even until day 7 of culture. Earlier on, Chu, H., et al. (2020) reported similar findings on the infectivity rate of SARS-CoV-2 on Calu-3 cell lines [11].

Consistent with previous reports, we found that MRC-5 and A549 are not susceptible to infection by all our SARS-CoV-2 isolates [11,19]. This could be due to the low expression of exogenous human ACE2 in these cells [13,20]. However, owing to the potential of these cells to be the model system for the discovery of antivirals targeting SARS-CoV-2 in humans, they have been successfully engineered to express human ACE2 and TMPRSS2 that are permissive to SARS-CoV-2 [9,12,21].

In this present study, we have selected SARS-CoV-2 isolates that were widely circulated among the Malaysian population during the second wave of SARS-CoV-2 in Malaysia, which took place from March to June 2020, of which lineage B.6 was dominant [22]. Among these isolates, whole genome analysis revealed mutations at the spike protein region for WC1114/20 and WI119/20. The mutations identified were R682Q (WC1114/20) and D614G (WI119/20). The R682Q mutation is a single spike protein mutation that occurs due to the Arg 682 substitution to Gln near the ‘furin-like’ S1/S2 cleavage site in the S protein [17]. Meanwhile, the D614G mutant has a glycine residue 614 (G614) substitution from aspartic acid (D614), which elevates virion spike density and infectivity [23]. Among the many mutations in SARS-CoV-2 identified during the pandemic, D614G is one of the most predominant in Malaysia and worldwide [22,24]. This mutation has been shown to increase viral entry, replication, and transmissibility of the virus by stabilizing the spike receptor binding protein (RBD), which in turn contributed to the stronger binding to the host cell receptor ACE2 [25]. 

A comparison of the isolates’ infectivity on the mammalian cell lines revealed that WI119/20 multiplied the highest at the MOI of 1, followed by WC81849/20 and WC1114/20 (Figure 4). Our study also detected a slight propagation of WI119/20 and WC81849/20 in MRC-5 and A549 cell lines. As reported before, the D614G substitution in the spike region proved to contribute to the stronger binding and replication of SARS-CoV-2 in vitro [26]. The exceptional multiplication and infectivity rate of WI119/20 in our study demonstrated the advantage of the D614G mutation in enhancing the replication of this virus.

The propagation of SARS-CoV-2 is the first step in providing the capacity to learn about this virus and would be the key step into vaccine production that is based on the inactivation of whole virus particles. Therefore, in addition to the adeptness of the isolate to propagate well in culture, they must also withstand the selection pressure when the isolate is subjected to repetitive passage during the culture. For this study, we looked for cells that will support the propagation of SARS-CoV-2 without causing significant pressure for the isolate to adapt the S1/S2 region of the S protein, which will result in a major genetic mutation that shifts the lineage of the isolate entirely. As described earlier, whole genome sequencing was performed on the propagated isolates in each of these cell lines to compare the genetic polymorphisms against the reference genome (Severe Acute Respiratory Syndrome Coronavirus 2 isolate Wuhan-Hu-1; MN908947.3). Our analysis revealed the virus isolates acquired abundant genetic polymorphisms following adaptation to various mammalian cell lines, mostly single nucleotide polymorphisms (snp). Remarkably, there was not a single SNP detected in the WC1114/20 isolate that was adapted into Vero CCL-81 when compared with the first passage, thus suggesting the compatibility of these cell lines in propagating the Lineage B Wuhan isolate. 

In summary, our study has successfully described the method for isolation and propagation of SARS-CoV-2 isolated in Malaysia. This study has generated information on the growth profile of this virus, which can serve as the basis for the propagation of this virus for various purposes, such as anti-viral studies and vaccine production.

## Figures and Tables

**Table 1:** Mapping statistics.

Isolate	Cell Lines	Number of Reads	Reads Mapped	% Map	Mean Coverage
WC1114/20	Vero E6	141,030	123,081	87.27292	945.8
	Vero CCL-81 *	124,444	107,571	86.44129	770.2
	Calu-3	143,562	113,939	79.36571	897.4
	MRC-5	278,926	201,393	72.20302	1426.5
	A549	102,394	93,037	90.86177	681
WI119/20	Vero E6	83,218	71,315	85.6966	508
	Vero CCL-81	100,388	83,888	83.56377	590.3
	Calu-3	117,250	105,248	89.76375	765.8
	MRC-5	85,116	74,421	87.43479	568
	A549	89,764	82,938	92.39562	629.6
WC81849/20	Vero E6	133,292	108,130	81.12265	757.2
	Vero CCL-81	66,516	56,280	84.61122	408.3
	Calu-3	156,780	133,842	85.36931	976.2
	MRC-5	220,494	177,962	80.71059	1279.9
	A549	83,086	78,500	94.48042	593.6

Reads mapped/% mapped represent the total number of reads that were mapped to the reference genome (MN908947.3). The mean coverage represents the per-base coverage of the total mapped reads against the reference genome. * No single nucleotide polymorphism (snp) was identified in WC1114/20 propagated in Vero CCL-81.
